# Management of sleep disturbance related to Alzheimer disease and dementia: An updated review of ClinicalTrials.gov

**DOI:** 10.1097/MD.0000000000043725

**Published:** 2025-08-08

**Authors:** Mohammed M. Aldurdunji

**Affiliations:** a Pharmaceutical Practices Department, College of Pharmacy, Umm Al-Qura University, Makkah, Saudi Arabia.

**Keywords:** Alzheimer disease, clinical trials, dementia, pharmacotherapy, sleep disturbance

## Abstract

Sleep disturbances are prevalent and commonly associated with Alzheimer disease (AD) and other forms of dementia, significantly impacting the quality of life for patients and their caregivers. These disturbances not only exacerbate cognitive decline but also contribute to the overall progression of neurodegenerative diseases. This review highlights the importance of managing sleep disturbances in patients with AD and dementia by analyzing completed clinical trials on this topic. A comprehensive search of the ClinicalTrials.gov database was performed on July 21, 2024, to identify all relevant clinical trials evaluating the efficacy of interventions for managing sleep disturbances in this population. Trials were included if they focused specifically on interventions for sleep disturbances in patients diagnosed with AD or dementia, were completed, and had results available. A total of 9 interventional and completed clinical trials related to sleep disturbance and AD were identified, involving 1139 participants. Three of the studies were nonpharmacological interventions, while 6 were pharmacological. Four studies were phase 2 clinical trials, and 1 was phase 3; the remaining were categorized as nonpharmacological. All studies were completed and had reported results. This review identifies a limited number of clinical trials addressing sleep disturbances in AD and dementia using either pharmacological or nonpharmacological interventions. Further clinical trials are recommended due to the impact of sleep disturbances on the quality of life for both patients and caregivers.

## 1. Introduction

Sleep disturbances are a common and well-documented feature of Alzheimer disease (AD) and other forms of dementia, negatively affecting both patients’ quality of life and their caregivers’ well-being.^[1]^ These disturbances not only exacerbate cognitive decline but also contribute to the overall progression of neurodegenerative diseases.^[2]^ The bidirectional relationship between sleep disorders and AD suggests that effectively managing sleep could help mitigate some of the cognitive and behavioral symptoms associated with the disease.^[3]^

Among the symptoms of AD, sleep-related issues are highly prevalent, affecting up to 60% of patients.^[4]^ Symptoms such as prolonged sleep latency, frequent awakenings, decreased total sleep time (TST), and impaired sleep efficiency often appear early and worsen as the disease progresses.^[5]^ Disruptions in circadian rhythms, such as a reversal of the sleep-wake cycle, are also common, likely due to the impact of AD pathology on the suprachiasmatic nucleus (SCN), the brain’s “master clock.”^[6]^ This disruption weakens circadian signals, resulting in irregular sleep-wake patterns. Patients may experience “sundowning,” where confusion and agitation increase in the late afternoon and evening, further complicating sleep patterns.^[6]^ AD is also associated with a significant reduction in restorative slow-wave sleep and increased wakefulness after sleep onset, leading to fragmented sleep, daytime sleepiness, and worsening cognitive dysfunction.^[7]^

There is growing evidence that sleep disturbances may accelerate cognitive decline in AD,^[8–10]^ creating a vicious cycle where poor sleep exacerbates cognitive symptoms, which in turn further disrupts sleep patterns. In addition to affecting patients, these disturbances place a considerable burden on caregivers, who often face stress, sleep deprivation, and burnout from managing nighttime disruptions and agitation.^[1]^ This can lead to caregiver burnout, increased stress, and a higher likelihood of institutionalization for patients. Therefore, effective management of sleep disturbances in AD can improve quality of life for both patients and caregivers.

Several mechanisms have been proposed to explain the link between sleep disturbances and AD. One of the most significant is the role of sleep, particularly slow-wave sleep, in the clearance of amyloid-β (Aβ) from the brain.^[11,12]^ Aβ, which accumulates abnormally in AD patients, forms plaques that are a hallmark of the disease. During sleep, the brain’s glymphatic system is more active, promoting the clearance of Aβ and other metabolic waste products. Disrupted sleep impairs this clearance process, leading to increased Aβ accumulation and potentially accelerating AD progression. Additionally, neurotransmitter systems involved in sleep regulation, such as melatonin^[13]^ and the hypocretin/orexin system,^[14]^ are disrupted in AD, further contributing to sleep difficulties and increased daytime sleepiness.

Moreover, chronic sleep deprivation and disruptions in sleep patterns can lead to increased inflammation and oxidative stress, both of which are implicated in AD pathology.^[7,15]^ Elevated inflammatory markers and oxidative damage are commonly observed in AD patients, suggesting that poor sleep quality might contribute to these detrimental processes and create a feedback loop that exacerbates both sleep disturbances and neurodegeneration.

The aim of this review is to comprehensively evaluate the efficacy of various interventions for managing sleep disturbances in patients with AD and dementia. By synthesizing data from clinical trials, this review seeks to provide an in-depth analysis of both pharmacological and non-pharmacological treatments. The objective is to assess the effectiveness of these interventions in improving sleep quality, reducing nocturnal awakenings, and stabilizing circadian rhythms in AD patients. Ultimately, this review aims to identify the most promising strategies for mitigating sleep-related issues in AD, enhancing patient quality of life, and alleviating caregiver burden. Additionally, the review will highlight gaps in current research and propose directions for future studies to optimize the management of sleep disturbances in this vulnerable population.

## 2. Methods

### 2.1. Search strategy

A comprehensive search of the ClinicalTrials.gov database was performed on July 21, 2024, to identify all the relevant clinical trials evaluating the efficacy of interventions for managing sleep disturbances in patients with AD and dementia. The search terms “AD,” “AD,” “Familial AD,” “Alzheimers Disease,” “Alzheimer Dementia,” “Alzheimer Dementia,” “disease,” “Disorders,” “Diseases,” and “disorder” were used in conjunction with “sleep disturbance” to generate pertinent results from the website search engine.

### 2.2. Reviewing search results

The process for identifying appropriate clinical trials relied on specific details of interest in the research. The inclusion criteria for selecting relevant clinical trials were required to be Clinical trials focusing specifically on interventions for sleep disturbances in patients diagnosed with AD or dementia as well as that to be completed and had results available. Any study that was not focusing on the intervention of sleep disturbance in AD and dementia were excluded from the review.

### 2.3. Data extraction

The following information were gathered and downloaded from the clinical trials.gov database: Study title and URL, Study design (e.g., randomized controlled trial, open-label trial), Interventions used, Primary and secondary outcome measures, Study phase, sample size. clinical trials that did not focus on sleep disturbances as a major intervention in AD, or those without available results, were excluded from the research.

## 3. Results

### 3.1. Analysis of number of research registration

**Figure 1.**
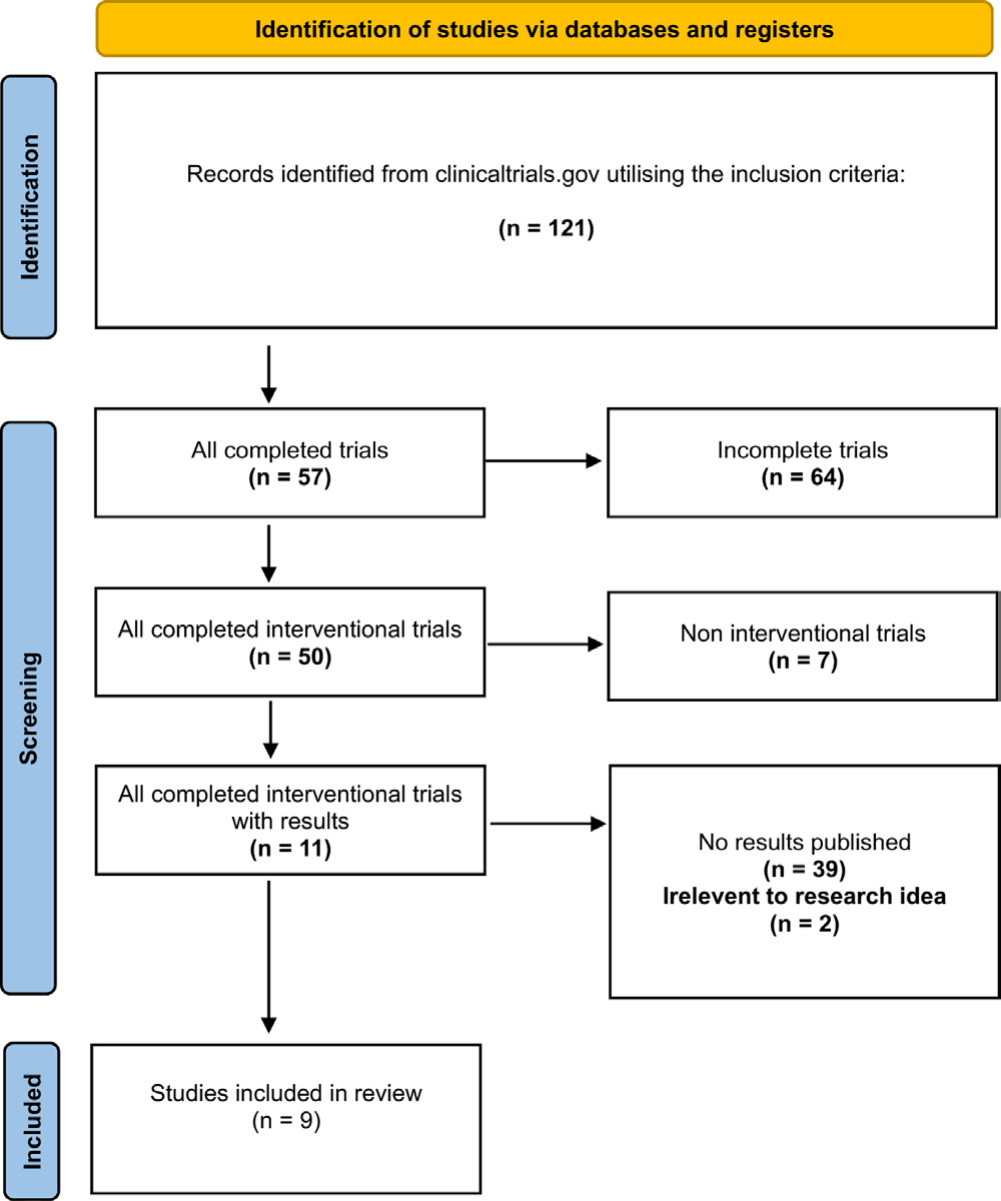
Flow diagram of trial selection process (updated from ClinicalTrials.gov July 21, 2024.).

A total of 9 study related to sleep disturbance and AD were found as an interventional and completed trials in the clinical trials.gov database while the remaining studies were excluded for not meeting the inclusion criteria (Fig. 1).

### 3.2. Trial characteristics

Of these 9 trials, there were a total of 1139 participants. All of the studies were interventional studies, completed and with reported results. The characteristics of the included clinical trials are summarized in Table 1. However, 3 of them were non-pharmacological intervention while the remaining 6 studies were pharmacological interventional studies. Furthermore, 4 studies were phase 2 clinical trials, and a single trial were phase 3, while the remaining studies (4 studies) were categorized as not applicable (a term used to describe trials without FDA-defined phases) which includes trials of devices or behavioral interventions. Finally, 8 of the 9 studies were randomized trials.

**Table 1:** Description of included clinical trials evaluating interventions for sleep disturbances in AD and dementia (ClinicalTrials.gov, accessed July 21, 2024).

	NCT number	Study title	Brief summary	Conditions	Interventions	Primary outcome measures	Secondary outcome measures	Age	Phases	Enrolment
1	NCT04533815^[16]^	Enhancing sleep quality for nursing home residents with dementia	-Pilot study for a full clinical trial (R33) to improve sleep in nursing home residents with ADRDs	-Alzheimer disease -Sleep disorder	Behavioral: LOCK sleep intervention	Total sleep time measured by actigraphy. For 15-wk sleep intervention period		- Adult - Older_adult	NA	23
2	NCT00940589^[17]^	Efficacy of circadinâ® 2 mg in patients with mild-to-moderate Alzheimer disease treated with AChE inhibitor	Evaluate the efficacy of Circadin® 2mg in patients with mild-to-moderate AD treated with an acetylcholinesterase (AChE) inhibitor. Assess impact on daytime somnolence	-AD -Sleep disorder	-Circadin -Placebo	Change from baseline to 24 wk in ADAS-cog (Alzheimer’s disease assessment scale- cognition) score. -ADAS-cog measures disturbances in memory, language, praxis, attention, and other cognitive abilities, often core symptoms of AD	Change from Baseline to 24 wk - IADL: measures independence in key life tasks - MMSE: Assesses cognitive function	- Adult - Older_adult	Phase2	73
3	NCT00814502^[18]^	Zolpidem CR and hospitalized patients with dementia	Compare the effectiveness of Zolpidem CR to placebo in improving sleep efficiency in hospitalized dementia patients.	-AD -Sleep disorder	-Zolpidem CR -placebo	-Sleep efficiency post-intervention, up to 3 wk. -Total Sleep minutes post-intervention, up to 3 weeks.	Measures of aggression, psychosis, general clinical status, cognitive measures, and mood symptoms RAGE: - DBRS - NPI - MADRS - MMSE - Time period	- Adult - Older_adult	NA	20
4	NCT00946530^[19]^	Light treatment for sleep/wake disturbances in AD	Demonstrate the efficacy of timed exposure to bright light for treating disturbed nighttime sleep and daytime wakefulness in community-dwelling dementia patients and their caregivers.	Sleep initiation and maintenance disorders -AD	-Device: bright light -Device: control	Total sleep time for 2 wk period	WASO	- Adult - Older_adult	NA	118
5	NCT03001557^[17]^	Study of LEM for irregular sleep-wake rhythm disorder and mild-to-moderate Alzheimer Disease Dementia	Determine the dose response of LEM on changes in actigraphy-derived sleep, wake, and circadian-rhythm related parameters.	-Irregular sleep-wake rhythm disorder -Alzheimer disease	-LEM 2.5, 5, 10, 15 mg -Matched placebo	–	–	–	–	–
6	NCT00843518^[20]^	Treatment for aggression and agitation in patients with AD	Determine whether the drug can help alleviate symptoms of aggression and agitation in participants with AD	AD	-LY451395 -Placebo	Primary outcome: mean change in NPI-4 A/A subscale score: Measures agitation/aggression, aberrant motor behavior, irritability/emotional lability, and disinhibition for 12 wk	Change from Baseline at wk 12 for: - NPI-10, NPI depression, NPI psychosis. - CMAI-C and CSDD. - FrSBe: frontal lobe behaviors - Cognitive dysfunction in AD	- Adult - Older_adult	Phase2	132
7	NCT02615002^[21]^	Safety and efficacy of piromelatine in mild AD Patients (ReCOGNITION)	Conduct a Phase 2, randomized, placebo-controlled, dose-ranging study of piromelatine (5, 20, and 50 mg daily for 6 months) versus placebo.	-AD -Sleep disorder	- Piromelatine -Placebo	Change in cNTB Z-Scores Global composite score combines z-scores from ISLT, OCL, IDN, DET, OBK, COWAT, and CFT tests. 26 wk measurement	CGIC - ADCS-MCI-ADL and ADAS-cog14 Safety and tolerability - Blood pressure - Heart rate - ECG interval results - Hematology - Blood chemistry	- Adult - Older_adult	Phase2	371
8	NCT02750306^[22]^	Safety and efficacy of suvorexant (MK-4305) for the treatment of insomnia in participants with AD (MK-4305-061)	Examine the safety and efficacy of suvorexant (MK-4305) to improve sleep in individuals with AD	-AD -Sleep disorder	-Suvorexant -Placebo	Change from baseline in TST Baseline and wk 4	Percentage of participants who: - Experienced 1 or more adverse events - Discontinued study drug due to an adverse event	- Adult - Older_adult	Phase3	285
9	NCT01482351^[23]^	MCI and OSA	Determine if CPAP treatment adherence predicts cognitive and everyday function after 1 yr in older adults with MCI, controlling for baseline differences.	Obstructive sleep apnea Mild cognitive Impairment	Behavioral: CPAP adherence intervention -BEHAVIORAL: Attention control intervention	Change from baseline at 6 mo and 1 yr for: HVLT-R, DS, MMSE and SCW.	Change from baseline at 6 mo and 1 yr - Sleep questionnaire (FOSQ) - Everyday Cognition (E-Cog) - AD cooperative study clinicians’ global -impression of change scale (ADCS-CGIC) - CDR Scale for	- Adult - Older_adult	NA	54

AD = Alzheimer disease, ADAS-Cog = Alzheimer disease assessment scale-cognition, ADRDs = Alzheimer disease or related dementias, CDR = clinical dementia rating, CGIC = change from baseline global-impression of change, DBRS = disruptive behavior rating scales, IADL = instrumental activities of daily living, LEM = lemborexant, MADRS = Montgomery-Asberg depression rating scale, MCI = mild cognitive impairment, MMSE = mini-mental state examination, NPI = neuropsychiatric inventory, OSA = obstructive sleep apnea, RAGE = rating scale for aggressive behavior in the elderly, TST = total sleep time, WASO = wake after sleep onset.

### 3.3. Sleep disturbance pharmacological management

#### 3.3.1. * Melatonin (circadin® 2 mg*)

The randomized, placebo-controlled trial titled “Efficacy of Circadin® 2 mg in Patients With Mild AD Who Suffer From Sleep Disorders” (NCT00940589),^[13]^ explored the impact of add-on prolonged-release melatonin (PRM) on cognitive functioning and sleep quality in 80 patients with mild-to-moderate AD, particularly those with insomnia. The primary outcome measure was the change in sleep quality from baseline to 24 weeks. The participants were treated with either 2 mg of PRM or placebo nightly for 24 weeks, following a 2-week placebo run-in period, in a randomized, double-blind, parallel-group design. Cognitive performance was assessed using the AD assessment scale-cognition (ADAS-Cog), instrumental activities of daily living (IADL), and mini-mental state examination (MMSE), while sleep was evaluated with the Pittsburgh sleep quality index (PSQI) and daily sleep diaries. The study found that PRM significantly improved cognitive performance as measured by the IADL and MMSE scores, and enhanced sleep efficiency compared to placebo. In the subgroup with comorbid insomnia, PRM showed more pronounced benefits in cognitive and sleep measures, with significant differences in IADL, MMSE, and PSQI scores. These results suggest that PRM group had a significant improvement compared to the placebo group (*P* < .05) on cognitive functioning and sleep maintenance, indicating a possible link between poor sleep and cognitive decline. PRM was well-tolerated with a safety profile comparable to placebo.

#### 3.3.2. Zolpidem CR

The study “Zolpidem CR and Hospitalized Patients With Dementia” (NCT00814502)^[18]^ investigated the efficacy of Zolpidem CR compared to placebo among hospitalized patients with Dementia of the AD experiencing sleep disturbances. the trial included 17 participants who received either Zolpidem CR 6.25 mg or a placebo after a 48-hour baseline measurement period. The primary outcome was measuring the total sleep minutes during the night. The results showed a least squares mean of 443.71 minutes (SE = 30.11) for the Zolpidem CR group and 422.49 minutes (SE = 29.27) for the placebo group, suggesting Zolpidem CR may improve sleep efficiency slightly more than placebo in this population However, some patients reported mild adverse effects such as dizziness and headache.

#### 3.3.3. Lemborexant

The study investigated the effects of lemborexant (LEM) on Irregular sleep–wake rhythm disorder in patients with mild-to-moderate AD dementia (AD-D).^[17]^ In the Core Phase, subjects aged 60 to 90 years were randomized to receive either placebo or LEM in doses of 2.5 mg, 5 mg, 10 mg, or 15 mg for 28 days. During the extension phase, eligible subjects (n = 25) initially received open-label LEM10, with the option to adjust the dose to LEM5 or LEM15. Treatment-emergent adverse events (TEAEs) were reported in 64% of subjects, with serious TEAEs in 12%, but no deaths. Common TEAEs included nasopharyngitis (16%), falls (12%), and somnolence (12%). Subjects on a modal dose of LEM10 (n = 17) showed a mean SDI total score (SDI-ts) improvement from 0.72 at baseline to −0.14 at Day 29, −0.37 at Day 133, and −0.12 at Day 223, indicating moderate improvement over time. LEM was well-tolerated over 1 to 14 months.

#### 3.3.4. Mibampator (LY451395)

The randomized, double-blind, placebo-controlled Phase 2 trial evaluated mibampator,^[20]^ an AMPA receptor potentiator, for treating agitation and aggression (A/A) in 132 outpatients with AD. Over 12 weeks, patients received either 3 mg of mibampator or placebo. The primary measure, the NPI-4-A/A subscale, showed no significant difference between groups, though both improved. Sleep disturbances were not considered as primary outcome for the trial however, it was considered in the neuropsychiatric symptom analysis. Overall, the study did not find significant efficacy of mibampator over placebo in managing sleep disturbances specifically.

#### 3.3.5. Piromelatine

This randomized, placebo-controlled, dose-ranging study (ReCognition) assessed piromelatine,^[21]^ a novel melatonin and serotonin receptor agonist, in participants with mild AD. Over 6 months, 371 participants aged 60 to 85 received 5, 20, or 50 mg of piromelatine daily or a placebo. Primary and secondary outcomes, including the computerized neuropsychological test battery (cNTB) and the PSQI, showed no statistically significant differences between piromelatine and placebo groups, nor were there safety concerns. However, genome-wide association studies (GWAS) revealed that a specific polymorphism cluster at 2q12 (5–6 SNPs) might predict piromelatine’s efficacy. Carriers of this polymorphism cluster (27% of the sample) showed significant cognitive improvement on cNTB but worsened on ADAS-Cog14 and PSQI with piromelatine. Noncarriers showed significant improvement on ADAS-Cog14 and PSQI with piromelatine. These findings suggest genetic markers could help identify patients who would benefit from piromelatine, warranting further investigation in larger clinical trials. The study highlighted the need for personalized approaches in treating mild AD and suggested the potential role of piromelatine in improving sleep quality and cognition, especially in noncarriers of the 2q12 polymorphism cluster.

#### 3.3.6. Suvorexant

This randomized, double-blind, placebo-controlled, 4-week trial assessed the efficacy and safety of suvorexant,^[22]^ an orexin receptor antagonist, for treating insomnia in patients with mild-to-moderate probable AD dementia. The trial involved 285 participants, with 142 receiving suvorexant (10 mg, increased to 20 mg if needed) and 143 receiving placebo. The primary endpoint was the change in TST from baseline, measured by polysomnography (PSG). Secondary measures included wake after sleep onset (WASO) and other sleep parameters. Results showed that suvorexant significantly improved TST by 28 minutes compared to placebo (*P* < .01) and reduced WASO by 16 minutes (*P* < .05). The improvement was particularly notable in the last third of the night. Suvorexant was well-tolerated, with somnolence being the most common adverse event. There was no significant worsening of cognitive function or neuropsychiatric symptoms. The study concluded that suvorexant effectively improved sleep in patients with AD dementia and insomnia, suggesting it could be a valuable treatment option for this population.

### 3.4. Sleep disturbance non-pharmacological management

#### 3.4.1. Behavioral therapies (leveraging outcomes for cognitive and knowledge translation) sleep intervention

The “Enhancing Sleep Quality for Nursing Home Residents with AD and Related Dementias” study (NCT04533815^[16]^) examined the impact of a behavioral intervention called leveraging outcomes for cognitive and knowledge translation (LOCK) on sleep quality among nursing home residents, particularly those with AD and related dementias. A total of 23 participants were included in the study in a single group intervention for a period of 15 weeks, the reported results showed a significant improvement in TST and sleep efficiency as measured by actigraphy (*P* < .01). Caregivers also reported a reduction in the severity of nighttime behaviors and an overall enhancement in daytime functioning of patients.

#### 3.4.2. Continuous positive airway pressure

This quasi-experimental pilot clinical trial investigated the impact of continuous positive airway pressure (CPAP) adherence on cognitive decline over 1 year in older adults with mild cognitive impairment (MCI) and obstructive sleep apnea (OSA).^[23]^ The study involved 54 participants aged 55 to 89 years, divided into 2 groups: CPAP adherent (≥4 hours per night, n = 29) and non-adherent (<4 hours per night, n = 25). Primary cognitive outcomes included memory and psychomotor/cognitive processing speed, measured using the Hopkins verbal learning test-revised (HVLT-R) and digit symbol subtests. Secondary outcomes included attention, daytime sleepiness, everyday function, and global cognition. Results showed significant improvements in psychomotor/cognitive processing speed in the CPAP adherent group compared to the non-adherent group, with a 1-year effect size of 1.25. Additionally, there were small to moderate effect sizes for memory, attention, daytime sleepiness, and everyday function favoring the adherent group. The study concluded that CPAP adherence significantly improves cognition in older adults with MCI and OSA and may slow cognitive decline, suggesting the need for larger, adequately powered trials to confirm these findings.

#### 3.4.3. Environmental modifications (bright light therapy)

The study titled “Light Treatment for Sleep/Wake Disturbances in AD” (NCT00946530)^[19]^ assessed the efficacy of bright light therapy in improving sleep-wake disturbances. The intervention involved exposure to bright light versus a control condition. A total of 118 individuals with memory impairment and their caregivers were enrolled in this trial. The primary outcome measure was TST. The participants were exposed to either bright white light (∼4200 lux) or dim red light (∼90 lux) for 30 minutes each morning for 2 weeks, alongside sleep hygiene therapy. Results showed that care recipients experienced decreased time in bed and TST, along with reduced sleep efficiency and increased WASO, indicating diminished sleep quality. No significant improvements in subjective insomnia symptoms were noted for care recipients. However, caregivers showed improved WASO, sleep efficiency, sleepiness, insomnia symptoms, and depressive symptoms, likely due to the structured protocol and sleep hygiene therapy rather than the phototherapy itself. The study concluded that while the phototherapy regimen was not beneficial for care recipients, it did improve sleep and mood for caregivers.

## 4. Discussion

The comprehensive review of clinical trials investigating the management of sleep disturbances in AD and dementia highlights the complexity and multifaceted nature of these interventions. Sleep disturbances are a prevalent issue in AD, significantly impacting both patients and their caregivers. Addressing these disturbances through various pharmacological and non-pharmacological treatments has shown mixed results. The pharmacological treatments, such as melatonin (Circadin®), zolpidem CR, lemborexant, mibampator, piromelatine, and suvorexant, have been evaluated for their efficacy in improving sleep quality among AD patients. The trials varied in their outcomes, with some showing significant improvements in sleep parameters and cognitive functions, while others did not demonstrate notable benefits. For instance, the use melatonin in AD patients with insomnia significantly improved cognitive performance and sleep efficiency. In contrast, the trial on mibampator did not find significant efficacy in managing sleep disturbances, despite considering them in the neuropsychiatric symptom analysis.

Non-pharmacological interventions, such as behavioral therapies, CPAP, and environmental modifications like bright light therapy, have also been explored. The LOCK sleep intervention, for example, demonstrated significant improvements in sleep quality and daytime functioning among nursing home residents with dementia. Similarly, CPAP adherence in older adults with MCI and OSA was associated with improved cognitive outcomes and reduced sleepiness, highlighting the potential cognitive benefits of treating sleep apnea in this population. However, the effectiveness of bright light therapy in improving sleep-wake disturbances showed limited benefits for care recipients, although it did improve mood and sleep parameters for caregivers.

Recent in vitro and preclinical studies have provided further insights into the mechanisms underlying sleep disturbances in AD and potential therapeutic targets. For example, studies on the role of the glymphatic system have revealed its critical function in clearing amyloid-β (Aβ) during sleep, suggesting that enhancing glymphatic clearance could mitigate AD pathology.^[11]^ Furthermore, experimental treatments aimed at enhancing slow-wave sleep, which is crucial for glymphatic function, have shown promise in animal models.^[24]^ Additionally, research on the orexin/hypocretin system, which regulates wakefulness, has led to the development of new orexin receptor antagonists that may offer benefits in treating insomnia associated with AD.^[14]^ Moreover, the involvement of aquaporin-4 (AQP4) in facilitating glymphatic clearance has been highlighted, with disruptions in AQP4 localization linked to impaired glymphatic function and increased Aβ accumulation.^[25]^

These findings underscore the importance of tailored interventions that consider individual patient characteristics, such as baseline cognitive status, comorbid conditions, and specific sleep disturbances. the co-utilization of the current innovative technologies in the wearable sleep-trackers, such as, smart watches, in monitoring sleeping patterns and adherence to interventions also represents a helpful tool to assist and accurately monitor the sleeping patterns for future research.^[26,27]^ Additionally, the integration of personalized medicine approaches, such as GWAS to identify genetic markers predicting treatment response, could further enhance the effectiveness of these interventions.

The review highlights several limitations in the analysis of sleep disturbance interventions in AD. Variability in intervention implementation fidelity across studies affects the consistency and comparability of outcomes. Few of the reported trial results were only present in ClinicalTrials.gov database, Incorrect data recording or a potential misclassification in the database can cause significant challenges, skewing results and interpretations. Future research should focus on conducting rigorous, well-designed trials with standardized protocols to ensure high fidelity in intervention implementation. Comprehensive data verification processes are essential to minimize misclassification errors. Longitudinal studies with larger sample sizes are needed to capture long-term effects and improve the reliability of findings. Despite these limitations, our analysis is unique and important, providing researchers and clinicians with valuable insights into the management of sleep disturbances in AD, ultimately enhancing treatment approaches and patient outcomes

## 5. Conclusion

The management of sleep disturbances in AD and dementia remains a critical aspect of improving patient quality of life and alleviating caregiver burden. While pharmacological treatments offer some benefits, non-pharmacological interventions and personalized approaches hold significant promise. Continued research, including preclinical studies and real-world trials, is essential to refine these interventions and ensure their effective implementation in diverse care settings.

## Author contributions

**Conceptualization:** Mohammed M. Aldurdunji.

**Methodology:** Mohammed M. Aldurdunji.

**Data interpretation:** Mohammed M. Aldurdunji.

**Literature review:** Mohammed M. Aldurdunji.

**Writing – original draft:** Mohammed M. Aldurdunji.
